# The rare hemoglobin variants Hb O-Arab and Hb D-Punjab identified in population-based genetic screening throughout Guangxi, China

**DOI:** 10.3389/fgene.2025.1622391

**Published:** 2025-08-14

**Authors:** Chunrong Gui, Zifeng Cheng, Yongsheng Chen, Yunting Ma, Hongfei Chen, Wei Wei, Xianda Wei, Juliang Liu, Xu Zhou, Qianqian Du, Yinghui Lai, Baoheng Gui

**Affiliations:** ^1^ Center for Medical Genetics and Genomics, The Second Affiliated Hospital of Guangxi Medical University, Nanning, China; ^2^ The Guangxi Health Commission Key Laboratory of Medical Genetics and Genomics, The Second Affiliated Hospital of Guangxi Medical University, Nanning, China; ^3^ The Second School of Medicine, Guangxi Medical University, Nanning, China; ^4^ Department of Hematology, The Second Affiliated Hospital of Guangxi Medical University, Nanning, China; ^5^ Berry Genomics Corporation, Beijing, China

**Keywords:** Hb O-Arab, Hb D-Punjab, hemoglobinopathy, genetic screening, Chinese population, single-molecule real-time sequencing, genotype-phenotype correlation

## Abstract

**Background:**

Hemoglobinopathies are a group of autosomal recessive disorders characterized by a high degree of clinical and genetic heterogeneity. Comprehensive genetic screening for hemoglobin variants is crucial for prevention and treatment of these conditions. Single-molecule real-time (SMRT) sequencing enables efficient and reliable analysis of common and complex or rare hemoglobin variants.

**Methods:**

We launched a population-based genetic screening program for hemoglobinopathies in Guangxi, China, using SMRT. The *in silico* structural predictions based on Alphafold2 were performed for the rare variants identified. Additionally, a comprehensive literature review was conducted to elucidate the origin and genotype-phenotype correlation of these variants.

**Results:**

A total of 11,019 participants throughout Guangxi were recruited via the screening program. In two unrelated families, the variants, Hb O-Arab and Hb D-Punjab at the same genetic locus, were identified with an extremely low frequency of 0.0045% [1/(11,019*2), respectively] in the population. Structural prediction showed Hb O-Arab exerted a relatively significant impact on the hemoglobin structure, whereas the influence of Hb D-Punjab was minimal. This was consistent with findings from the literature review and the two recruited families, which confirmed that individuals with Hb O-Arab presented relatively obvious manifestations compared to those with Hb D-Punjab.

**Conclusion:**

Two rare variants, Hb O-Arab and Hb D-Punjab, were identified in Guangxi, China using SMRT. The first report of Hb O-Arab enriches the spectrum of hemoglobin variants in the Chinese population. Analyzing the frequency, origin and genotype-phenotype correlation of these variants could pave the way for clinical management and genetic counseling for hemoglobinopathies.

## Introduction

Hemoglobinopathies are a group of inherited blood disorders that are classified as autosomal recessive genetic hemolytic anemias. These conditions stem from a variety of hemoglobin (Hb) variants, which often result from mutations or deletions within the genes encoding the α- or β-globin chains of hemoglobin. The genetic alterations can lead to either a reduced production of these globin chains or structural changes in the hemoglobin molecule itself. When there is a reduced production of these globin chains, the resulting conditions are collectively known as thalassemia syndromes. Structural changes in hemoglobin cause abnormal hemoglobin, such as Hb S, Hb E, Hb O, and Hb D (Harteveld et al., 2022). Globally, there are approximately 350 million carriers of thalassemia, and over 300,000 newborns are affected by hemoglobinopathies, such as sickle cell anemia or thalassemia, etc. each year (Modell et al., 2008; Weatherall, 2010). Southern China has a particularly high prevalence of thalassemia, with Guangxi Province being a hotspot, where the prevalence rate approaches 20% (Lai et al., 2017). The clinical manifestation of hemoglobinopathies varies widely, from asymptomatic carriers to severe, life-threatening conditions (Kohne, 2011). Heterozygous carriers of these variants are typically asymptomatic but may exhibit hematological characteristics. However, homozygous or compound heterozygous states can result in clinically significant phenotypes with varying degrees of severity, such as thalassemia major, thalassemia intermedia, sickle cell syndrome, and Hb E syndrome.

Hemoglobinopathies are characterized by a remarkable level of genetic diversity. To navigate this genetic landscape, specialized databases like HbVAR and ithanet have been created to document and manage numerous genetic variations associated with hemoglobinopathies (Giardine et al., 2021; Kountouris et al., 2014). The HbVAR database, in particular, has amassed an extensive catalog of over 1800 distinct variants involving hemoglobin gene cluster, including *HBA1, HBA2*, and *HBB* (Giardine et al., 2021). Among these variants, changes at the 122nd codon of the β-globin gene, specifically c.364G>A [p.(Glu122Lys)] and c.364G>C [p.(Glu122Gln)], result in Hb O-Arab and Hb D-Punjab, respectively (van Gammeren et al., 2020). These distinct amino acid substitutions lead to different clinical manifestations and effects on red blood cell morphology and function. Both variants have a global distribution, with Hb D-Punjab being more prevalent in regions such as Punjab, India, and also found in Italy, Belgium, Austria, and Turkey (Torres et al., 2015). Hb O-Arab is found in people from the Middle East, and the Mediterranean (Elbashir and Elsayed Yousif, 2023).

Given the high genetic heterogeneity and the complexity of variations in hemoglobinopathies, there is an urgent need for an efficient and reliable method for screening and diagnosing of the hemoglobinopathies. Recently, a cutting-edge approach based on single-molecule real-time (SMRT) sequencing targeting the hemoglobin gene cluster has emerged (Xu et al., 2020). Benefiting from its long-read sequencing, the SMRT method comprehensively encompasses the full spectrum of known structural variations, single nucleotide variants (SNVs), and insertions/deletions (InDels) involving the *HBA1*, *HBA2*, and *HBB* gene clusters. Rigorous retrospective and prospective multi-center cohort analyses have demonstrated its efficiency and reliability in identifying and analyzing the common and even complex or rare hemoglobin variations, enabling accurate diagnosis and comprehensive understanding of hemoglobinopathies (Xu et al., 2020; Liang et al., 2021).

In this study, we launched a population-based genetic screening initiative for hemoglobinopathies in Guangxi, China, using the SMRT sequencing. Two rare hemoglobin variants, Hb O-Arab and Hb D-Punjab, were identified, whose impact on the structure of the hemoglobin were predicted. Furthermore, we conducted a literature review to analyse the origins and genotype-phenotype correlations of the two variants.

## Materials and methods

### Population and subjects

A population-based genetic screening project for hemoglobinopathies was conducted throughout Guangxi Zhuang Autonomous Region, China, spanning from July 2021 to December 2024. Physical examination and clinical assessment were performed for the subjects and their peripheral blood samples were collected for further hematological screening and genetic analysis.

### Hematological screening

Routine hematological indicators were measured by automatic hematological analyzer (LH780, Beckman Co., Nanning, China or BC-6000, Mindray Co., Nanning, China), and standard hemoglobin testing was performed by automatic high-pressure liquid-flow capillary electrophoresis (CAPILLARYS2, Sebia Co., Nanning, China), according to the manufacturer’s instructions. Normal reference ranges of the hematological indicators included mean corpuscular volume (MCV)≥82 fL, mean corpuscular Hb (MCH) ≥27 pg, Hb A_2_ levels between 2.4% and 3.5%, and Hb F ≤ 2%.

### Genetic screening by the SMRT sequencing

The SMRT sequencing was conducted as previously described (Xu et al., 2020; Liang et al., 2021). Briefly, genomic DNA was extracted from peripheral blood and subjected to multiple long range PCR to amplify the hemoglobin gene cluster including the *HBA1*, *HBA2*, *HBB*, etc. The amplified products were input for library preparation and subsequent sequencing on a PacBio Sequel II SMRT sequencer (Pacific Biosciences Inc., Menlo Park, United States), following the manufacturer’s instructions. The generated raw subreads were subsequently processed using circular consensus sequencing software (RRID: SCR_021174, Pacific Biosciences Inc., Menlo Park, United States) and the Pbbioconda package (Pacific Biosciences Inc., Menlo Park, United States) to obtain circular consensus sequencing reads, which were mapped to the GRCh38 reference genome and further used for variant calling using FreeBayes1.3.4 (RRID: SCR_010761, https://www.geneious.com/plugins/freebayes). The pathogenicity of the candidate variants was classified according to the ACMG/AMP guidelines (Richards et al., 2015) and information documented in hemoglobin variant databases, such as HbVar (https://globin.bx.psu.edu/hbvar/), ithanet (https://www.ithanet.eu/), and LOVD (https://www.lovd.nl/).

### Validation of the candidate variants identified by the SMRT sequencing

Sanger sequencing was employed to verify SNVs and InDels, followed by locus-specific amplification. Multiplex ligation-dependent probe amplification (MRC Holland, Amsterdam, Netherlands) was conducted to confirm structural rearrangements, including large deletions or duplications within specific hemoglobin gene regions. Additionally, gap polymerase chain reaction (Gap-PCR) (Yilifang Bio, Shenzhen, China) was utilized to detect hotspot deletions in the Chinese population, targeting--^SEA^, -α^3.7^, -α^4.2^, and--^THAI^, according to the manufacturer’s protocol. A sample containing the known heterozygous -α^3.7^ variant was used as the positive control. Meanwhile, a sample without any known *HBA1* or *HBA2* variants, as well as nuclease-free water, were used as the negative and blank controls, respectively. The DNA marker was included in the kit, and an amplification fragment of 1.7 kb indicated the presence of the internal control sequence.

### Structural prediction and visualization

The hemoglobin structures of the wild-type and mutant forms, including Hb O-Arab and Hb D-Punjab, were predicted using the ColabFold v1.5.5 (RRID: SCR_025453, https://colab.research.google.com/github/sokrypton/ColabFold/blob/main/AlphaFold2.ipynb), an online platform for AlphaFold2. The predicted local distance difference test (pLDDT) score above 70 was considered with high confidence. All models were visualized using PyMOL (RRID: SCR_000305), with hydrogen bonds displayed using the default settings.

### Retrospective analysis of similar cases reported in the literature

The literature search was performed to summarize the hematological features and clinical symptoms of individuals with the rare variants, Hb O-Arab and Hb D-Punjab. The literature search was conducted in the PubMed, Web of Science, and National Center for Biotechnology Information (NCBI) databases through keywords “hemoglobin O Arab”, “Hemoglobin O Arab”, “Hb O-Arab”, “hemoglobin D-Punjab”, “Hemoglobin D-Punjab”, “Hb D-Punjab”. Titles and abstracts selected from the initial search were first scanned, and the full papers of potentially eligible studies were reviewed. Articles were excluded for the following reasons: 1) the articles were not in English; 2) the full version of the articles were not available; 3) the articles did not report the hematological characteristics and clinical manifestations.

## Results

A total of 11,019 participants from throughout Guangxi were recruited via the genetic screening program for hemoglobinopathies, with ages ranged from the neonatal period to 86 years old, including 2,673 children and adolescents, and 8,346 adults. Among these participants, there were 5,310 males and 5,709 females. Totally, 165 hemoglobin variants were identified, of which 83 were variants in the *HBB* gene. In two unrelated families, the hemoglobin variants, Hb O-Arab and Hb D-Punjab at the same genetic locus, were identified with an extremely low frequency of 0.0045% [1/(11,019*2), respectively] among the screened population.

### A rare compound heterozygous variant Hb O-Arab and β^0^-thalassemia in family 1

**FIGURE 1:**
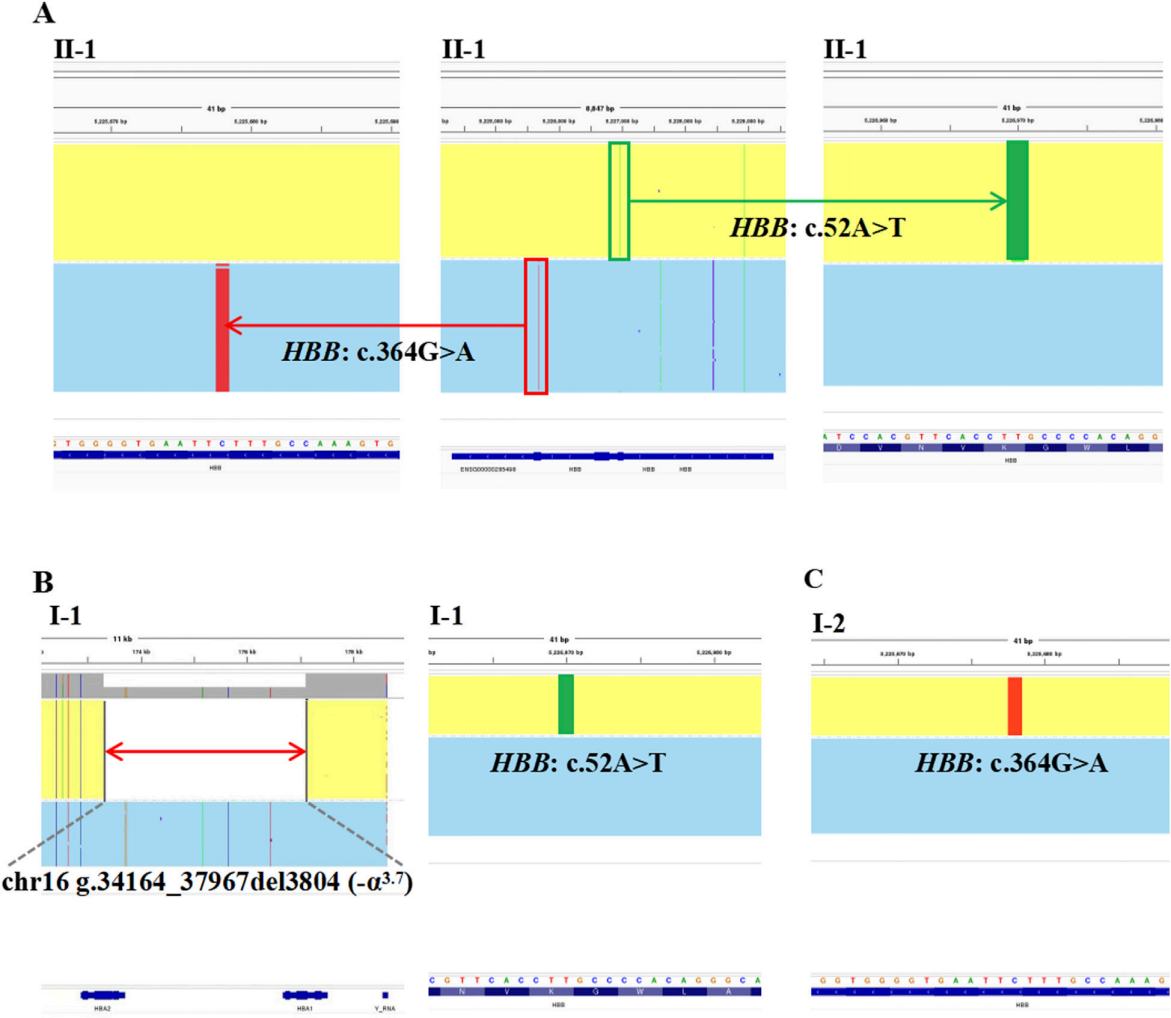
The variants identified by SMRT in Family 1. The integrative genomics viewer plots of the variants detected by SMRT in Family 1. **(A)** The variants, *HBB*: c.364G>A, co-occurring with *HBB*: c.52A>T, in II-1. **(B)** The deletion, chr16: g.34164_37967del3804 (−α^3.7^) and the variant, *HBB*: c.52A>T, in I-1. **(C)** The variant, *HBB*: c.364G>A, in I-2.

**FIGURE 2:**
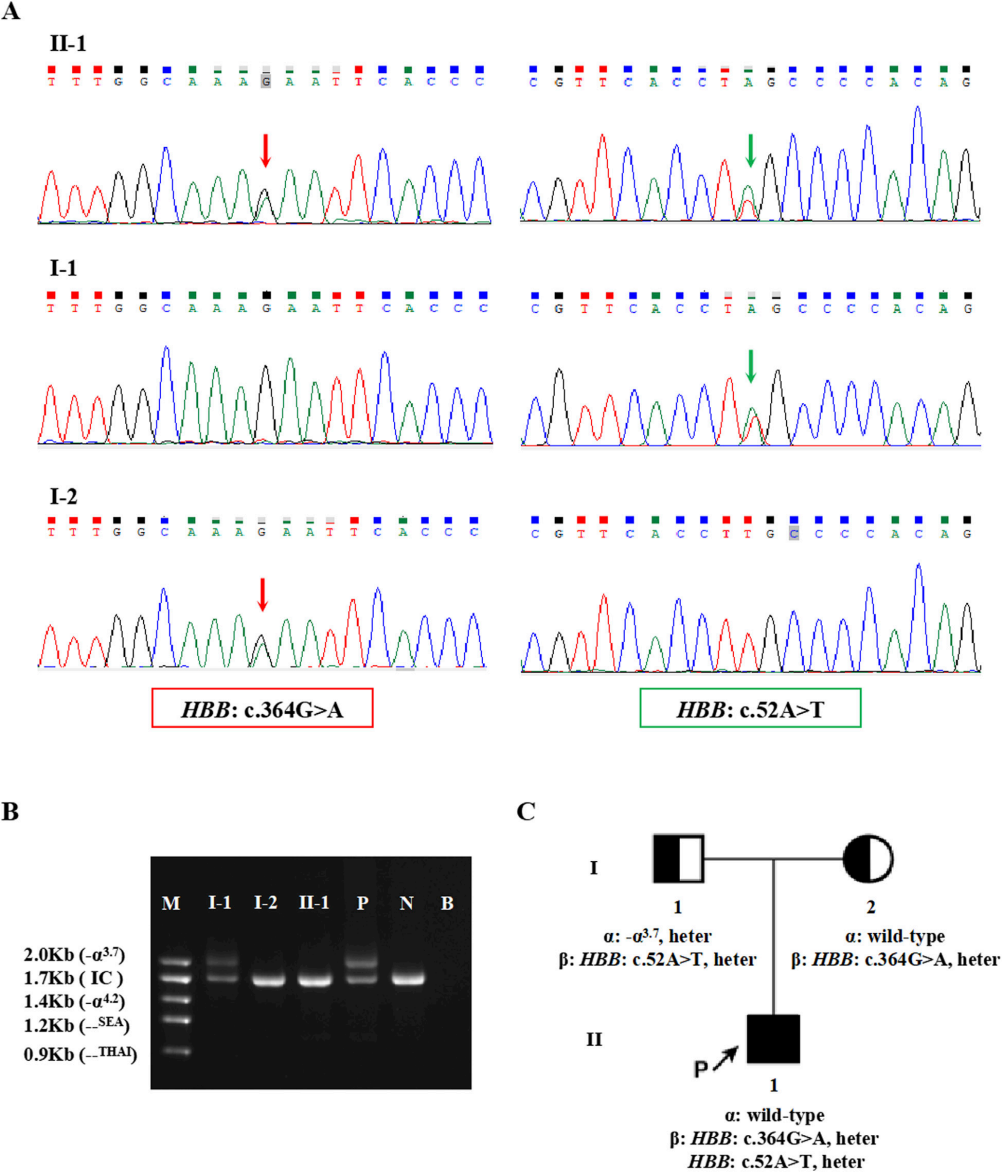
The variants validated by conventional approaches in Family 1. **(A)** The results of Sanger sequencing in Family 1. The red arrows indicate the variant, *HBB*: c.364G>A, and the green arrows indicate the variant, *HBB*: c.52A>T. **(B)** Agarose gel electrophoresis analysis for the Gap-PCR products in Family 1. M: DNA marker, P: positive control, N: negative control, B: blank control, IC: internal control. **(C)** Pedigree chart of Family 1.

The proband in Family 1, an 18-year-old Chinese man, visited our hospital with complaints of increased bilirubin, icteric sclera, and skin, but without subcutaneous bleeding, which indicated possible hemolytic anemia. The SMRT sequencing detected substitutions at codons 17 [NM_000518.5(*HBB*): c.52A>T p.(Lys18Ter)] and 122 [NM_000518.5(*HBB*): c.364G>A p.(Glu122Lys)] of the *HBB* gene simultaneously (Figure 1A). These two variants were located *in trans* and presented in a compound heterozygous pattern. Family analysis showed that the variant c.52A>T was from the father and c.364G>A was from the mother (Figures 1B,C, 2C). Additionally, a hotspot heterozygous -α^3.7^ deletion (chr16: g.34164_37967del3804) was identified in the father (Figure 1B). The variants c. 52A>T and c. 364G>A were further confirmed by Sanger sequencing (Figure 2A) and the -α^3.7^ was validated by Gap-PCR (Figure 2B).

**FIGURE 3:**
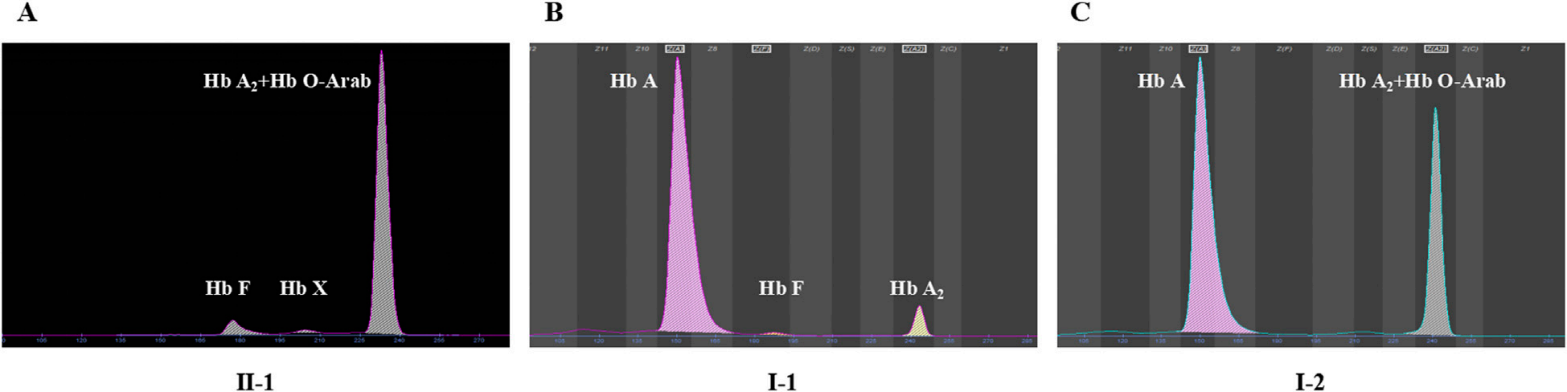
The capillary electrophoresis analysis of hemoglobin (Hb) in Family 1. **(A–C)** show the electrophoresis results of the proband (II-1), and his father (I-1) and mother (I-2) in Family 1, respectively. Specific hemoglobin peaks for Hb A, Hb A2, Hb F, and Hb O-Arab are displayed. Hb X: uncharacterized hemoglobin peak.

Among the two variants, c.52A>T also called CD17 (A>T) could cause β^0^-thalassemia, and c.364G>A also known as Hb O-Arab could alter the structure of hemoglobin. Thus, the heterozygosity of these two variants may explain the proband’s phenotype. This was confirmed by the routine hematological analysis, which showed significantly decreased MCV and MCH indicating microcytic hypochromic anemia (Table 1), together with abnormal hemoglobin presenting as an overlapping peak of Hb A_2_ and Hb O-Arab (92.5%), an Hb F peak (6.4%) and an uncharacterized Hb X peak (1.1%), but without the Hb A peak (Figure 3A). The parents also exhibited relatively low MCV and MCH levels (Table 1). An abnormal overlapping peak of Hb A_2_ and Hb O-Arab was also observed in his mother, while a mild increase in Hb A_2_ (5.4%) was detected in his father (Figures 3B,C).

**TABLE 1:** Hematological and electrophoretic characteristics in Family 1.

Parameters	Proband (II-1)^a^	Father (Ⅰ-1)^b^	Mother (Ⅰ-2)^b^	Reference range
RBC	5.46	7.19 ↑	5.68 ↑	4.3–5.8^a^/3.8–5.1^b^
HGB (g/L)	107.4 ↓	144.00	131.00	130–175^a^/115–150^b^
HCT	0.355 ↓	0.458	0.406	0.4–0.5^a^/0.35–0.45^b^
MCV (fL)	65.12 ↓	63.60 ↓	71.50 ↓	82–100
MCH (pg)	16.69 ↓	20.00 ↓	23.10 ↓	27–34
MCHC (g/L)	302.30 ↓	315.00 ↓	324.00	316–354
RDWCV	0.20 ↑	0.16 ↑	0.16 ↑	0.115–0.145
PLT	208.70	257.00	306.00	125–350
PCT	0.251	0.278	0.430 ↑	0.11–0.28
MPV (fl)	12.05 ↑	10.80	14.10 ↑	7–11
PDW	0.18 ↑	0.16	0.16	0.15–0.17
Hb A (%)^c^	0 ↓	93.8 ↓	65.9 ↓	94.5–97.6
Hb F (%)^c^	6.4 ↑	0.8	0	≤2
Hb A_2_ or Hb A_2_ + Hb O-Arab (%)^c^	92.5 ↑	5.4 ↑	34.1 ↑	2.4–3.5
Hb X (%)^c^	1.1	0	0	—

RBC, red blood cell count; HGB, hemoglobin; HCT, hematocrit; MCV, mean corpuscular volume; MCH, mean corpuscular hemoglobin; MCHC, mean corpuscular hemoglobin concentration; RDWCV: red blood cell distribution width coefficient of variation; PLT, platelet; PCT: plateletcrit; MPV, mean platelet volume; PDW, platelet distribution width; Hb X, uncharacterized hemoglobin peak.

^a^ These values were obtained using LH780 Hematology Analyzer.

^b^ These values were obtained using BC-6000, Hematology Analyzer.

^c^ These values were obtained using CAPILLARYS2 automatic high-pressure liquid-flow capillary electrophoresis.

### A rare heterozygous variant Hb D-Punjab in family 2

**FIGURE 4:**
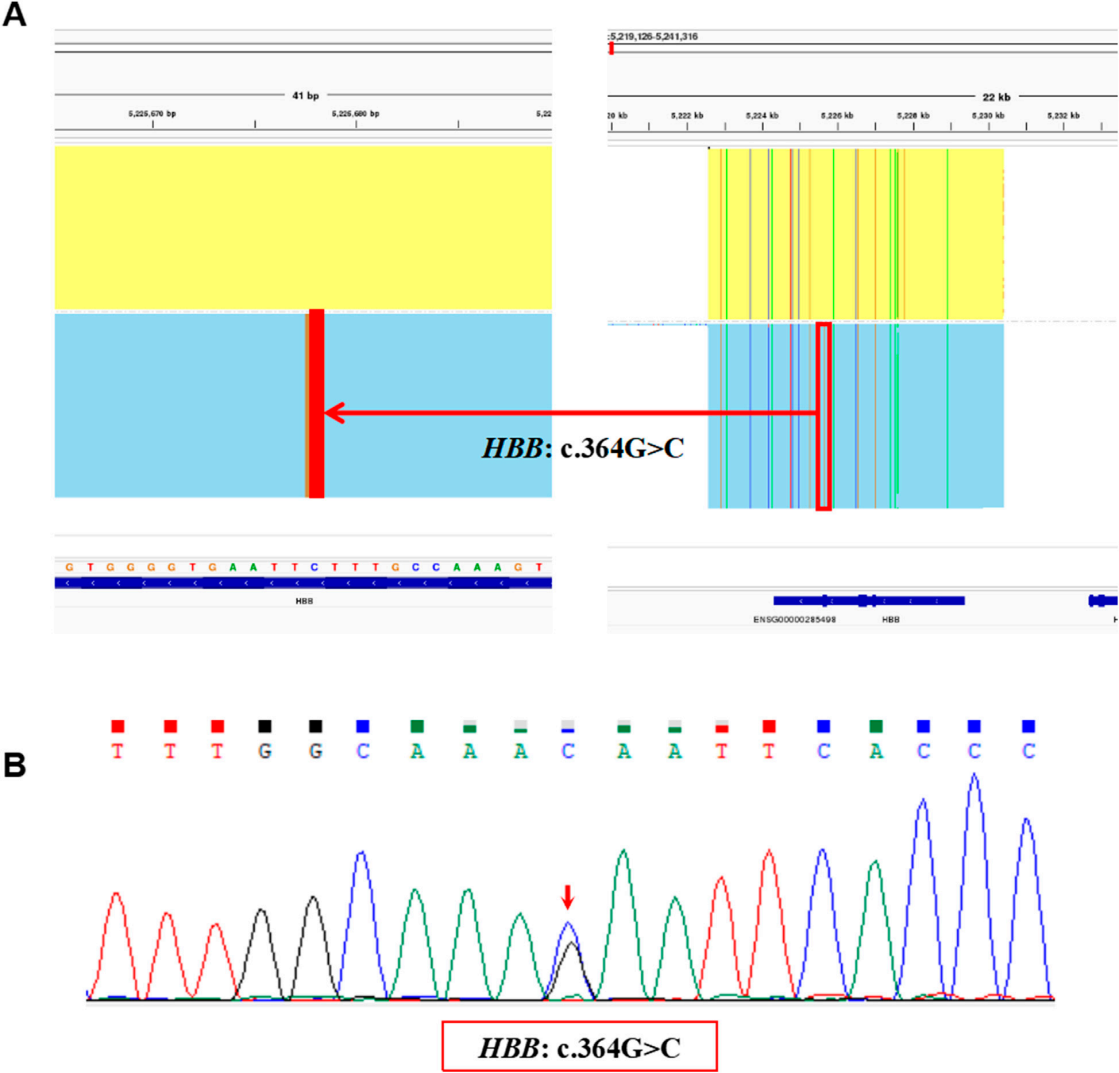
The variants identified by SMRT and validated by Sanger sequencing in Family 2. **(A)** The integrative genomics viewer plot of the variant, *HBB*: c.364G>C, detected by SMRT in Family 2. **(B)** Sequentially, Sanger sequencing confirmed the presence of the variant, *HBB*: c.364G>C indicated by the red arrow.

The proband in Family 2 was a 4 years old boy and participated in the genetic screening program for hemoglobinopathies. A heterozygous variant, NM_000518.5(*HBB*): c.364G>C p.(Glu122Gln), also called Hb D-Punjab, was identified in the proband by the SMRT sequencing (Figure 4A) and was validated by Sanger sequencing (Figure 4B). It was a rare Hb variant at the same genetic locus as the Hb O-Arab. Unfortunately, the hematological results of the proband were unavailable and the family refused to blood resampling for further analysis and validation.

### Structural prediction and alterations of hydrogen bonds

**FIGURE 5:**
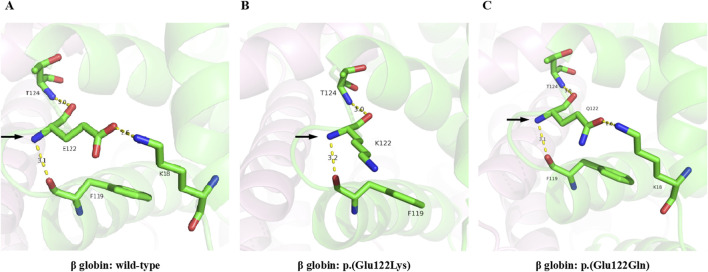
3D-structure of Hb O-Arab and Hb D-Punjab compared with wild-type β globin. The 3D-structures of the wild-type β globin **(A)**, Hb O-Arab **(B)**, and Hb D-Punjab **(C)** were predicted by Alphafold2. The carbon, nitrogen, and oxygen atoms are colored green, blue, and red, respectively. The hydrogen bonds are displayed as yellow dashed lines.

The pLDDT scores of all the structures were above 70, which indicated that the predictions were highly confident. In wild-type hemoglobin, the glutamic acid at residue 122 showed hydrogen bonds with lysine 18 (2.6Å), phenylalanine 119 (3.1Å), and threonine 124 (3.0Å), respectively (Figure 5A). However, in Hb O-Arab, the substitution of glutamic acid by lysine at residue 122 decreased the number of hydrogen bonds with the surrounding residues, only interacting with phenylalanine 119 (3.2Å) and threonine 124 (3.0Å) (Figure 5B). The loss of hydrogen bonds may alter the stability of the protein, potentially resulting in the clinical manifestation of anemia. By contrast, in Hb D-Punjab, the substitution of glutamic acid by glutamine at residue 122 maintains interactions with lysine 18 (2.6Å), phenylalanine 119 (3.1Å), and threonine 124 (3.0Å) (Figure 5C). Both the number and length of these hydrogen bonds remained unchanged, which indicated Hb D-Punjab was unlikely to significantly alter the protein structure, further suggesting its effect on the phenotype may be minimal.

### Hematological characteristics and clinical manifestations of individuals carrying the variants

Individuals with Hb O-Arab showed various hematological features and clinical symptoms across different situations. Although the subjects with homozygous Hb O-Arab typically had mild to moderate anemia with a lower hemoglobin levels, most of them were asymptomatic, and only a few of them exhibited mild clinical symptoms such as lassitude, jaundice and splenomegaly (Dror, 2013; Nagel et al., 1999; Efremov et al., 1977; Heard et al., 1991). When the variant co-existed with Hb S, the corresponding cases usually presented with mild to moderate anemia, jaundice, and splenomegaly, as well as clinical characteristics similar to those with sickle cell disease (SCD), such as acute chest syndrome, recurrent vaso-occlusive painful events, dactylitis, hemolytic and so on (Nagel et al., 1999; Rachmilewitz et al., 1985; Zimmerman et al., 1999). Individuals carrying compound heterozygous Hb O-Arab and β-thalassemia variants typically manifested mild to moderate microcytic hypochromic anemia with reduced levels of MCV and MCH as well as an elevated level of Hb A_2_, similar to β-thalassemia traits. They were typically asymptomatic, and only a few individuals exhibited mild jaundice and splenomegaly. As expected, the hematological characteristics and clinical manifestations of individuals carrying compound heterozygous Hb O-Arab and β^0^-thalassemia variants were more severe than those of individuals with Hb O-Arab and β^+^-thalassemia variants (Rachmilewitz et al., 1985; Lacerra et al., 1993; Kalai et al., 2024; Morlé et al., 1984; Moumni et al., 2011; Nikolov et al., 1989). Detailed information of the reported cases was provided in Table 2.

**TABLE 2:** Hematological characteristics and clinical manifestations of cases with Hb O-Arab.

Country	Age (year)	HGB (g/dL)	MCV (fL)	MCH (pg)	Hb A (%)	Hb A_2_ (%)	Hb F (%)	Hb O-Arab (%)	Hb S (%)	Hb X (%)	Clinical manifestations	Reference
Hb O-Arab, homo												
Sudan	0.75–3	7.8–9.4	49–74.5	17.5–27.5	0	0–9.1	21–32.5	67.2–79	NA	NA	Asymptomatic	Dror (2013)
Tunisian	13	13.5	83.4	NA	NA	NA	1.4	96.4	NA	NA	Jaundice, splenomegaly, lassitude, anorexia, epigastric pain	Nagel et al. (1999)
Yugoslavia	18	11.7	NA	30	NA	NA	1.6	100	NA	NA	NA	Efremov et al. (1977)
Moroccan	20	10.8	81	26.2	NA	NA	NA	NA	NA	NA	Asymptomatic	Heard et al. (1991)
Hb S + Hb O-Arab, heter												
Israel	6–23	8.7–11.2	83–95	26–33	NA	NA	4.9–13.2	NA	NA	NA	Abdominal pain, jaundice, pneumonia, pleural effusion, ulcus cruris, fever, arthritis or NA	Rachmilewitz et al. (1985)
Tunisia	20–26	9.9–10	86.3–86.7	NA	NA	NA	7.3–7.4	45.3–46.3	43.8–44.9	NA	NA	Nagel et al. (1999)
African-American	2.7–62.5	6.1–9.9	64–94 or NA	NA	NA	NA	0.6–20.3 or NA	NA	NA	NA	Acute chest syndrome, cerebrovascular accident, dactylitis, developmental delay, aplastic crisis, pulmonary stenosis, sepsis, meningitis (death), vaso-occlusive crisis, chronic renal failure, gallstones, osteomyelitis, avascular necrosis, nephropathy, retinopathy, congestive heart failure, deep vein thrombosis, leg ulcers, pulmonary fibrosis, multiorgan failure (death)	Zimmerman et al. (1999)
Hb O-Arab + β^+^-thalassemia, heter												
Israel	10–23	9.7–10.9	66–87	17–27	14.9–18.9	NA	3.3–7.8	74.7–81.2	NA	NA	Asymptomatic	Rachmilewitz et al. (1985)
Albanian	17	10.7	87	27	8.5	3.5	NA	NA	NA	84	Jaundice, splenomegaly	Lacerra et al. (1993)
Hb O-Arab + β^0^-thalassemia, heter												
Tunisian	0.33–56	6.8–9	63.5–64.7	19.3–20.17	0 or NA	4.1 or NA	6.2–27.6	72.4–89.7	NA	NA	Neonatal jaundice, splenomegaly, fever	Kalai et al. (2024), Moumni et al. (2011)
Moroccan	33	10.4	69.2	22.2	0	NA	12.19	78.11	NA	NA	Splenomegaly, intermittent asthenia, jaundice	Morlé et al. (1984)
Italy	55	8.9	53	19	0	2.8	NA	NA	NA	96	Jaundice, splenomegaly	Lacerra et al. (1993)
Yugoslavia	26	10	68	26	0	0	15	NA	NA	85	Hepatosplenomegaly, occasional abdominal pains, malaise, fatigue	Nikolov et al. (1989)

Homo: homozygous; Heter: heterozygous; NA: Not Available. Hb X, uncharacterized hemoglobin peak.

The Hb D-Punjab mainly existed in three forms: Hb D-Punjab homozygous, Hb S combined with Hb D-Punjab, Hb D-Punjab combined with β-thalassemia. Individuals with homozygous Hb D-Punjab were typically asymptomatic with normal hematological characteristics (el-Kalla and Mathews, 1997; Silva-Pinto et al., 2014), although a few of them developed mild to moderate anemia and led to pallor and fatigability (Singh et al., 2023; Spandana et al., 2022). The association of this variant with Hb S or thalassemia also occurred. Usually, the compound heterozygous Hb D-Punjab and β-thalassemia caused mild microcytic and hypochromic anemia with reductions in MCV and MCH and elevated Hb A_2,_ but showed no clinical changes (el-Kalla and Mathews, 1997; Perea et al., 1999; Panyasai et al., 2017; Fucharoen et al., 2002). Occasionally, individuals with this profile experienced weakness, hepatosplenomegaly and jaundice (Shekhda et al., 2017). The compound heterozygosity for Hb S and Hb D-Punjab resulted in moderately severe anemia with a reduction of Hb levels, and in addition to jaundice and hepatosplenomegaly, these individuals also presented clinical symptoms similar to those of sickle cell disease (SCD). Pain due to vaso-occlusive crisis was one of the most common complications, and acute chest syndrome as well as acute splenic sequestration (Torres et al., 2016; Adekile et al., 2010), also occurred in cases of this form. Detailed information was provided in Table 3.

**TABLE 3:** Hematological characteristics and clinical manifestations of cases with Hb D-Punjab.

Country	Age (year)	HGB (g/dL)	MCV (fL)	MCH (pg)	Hb A (%)	Hb A_2_ (%)	Hb F (%)	Hb D-Punjab (%)	Hb S (%)	Hb X (%)	Clinical manifestations	Reference
Hb D-Punjab, homo												
India	13–62 or NA	6.3–15.3	60–79	17.6–27 or NA	0–30.1 or NA	1.1–3.3 or NA	0.2–10.6 or NA	55.7–96.6 or NA	NA	NA	Palpable spleen, jaundice, cholestatic hepatitis, fever, progressive pallor, fatiguability, generalized weakness, awareness of mass in the abdomen, icterus, joint pain, cough, breathlessness, severe anemia or asymptomatic or NA	Singh et al. (2023), Politis-Tsegos et al. (1975), Biswas and Pillai (2019), Shanthala Devi et al. (2016), Desai et al. (2003)
Iran	52	14.6	83.9	27.6	NA	2.8	NA	97	NA	NA	Asymptomatic	el-Kalla and Mathews (1997)
Brazil	41	13.7	82	29.4	NA	NA	NA	NA	NA	NA	Asymptomatic	Silva-Pinto et al. (2014)
Hb S + Hb D-Punjab, heter												
Nepal	29	7	99.9	37.1	NA	3.8	7.80	43.30	32.00	NA	Recurrent episodes of jaundice, episodic severe backache radiating to chest, fever, pallor	Ali et al. (2020)
Brazil	6–43 or NA	6.2–9.5 or NA	81 or NA	29 or NA	NA	1.8–4.2 or NA	0.6 or NA	35–50.3 or NA	33–48 or NA	NA	Recurrent painful crises, acute chest syndrome, avascular necrosis, leg ulcer, priapism, anemic, hand-foot syndrome, several episodes of abdominal pain, palpable liver or asymptomatic	Torres et al. (2016), Zago and Costa (1988), Rezende Pdo et al. (2016), Nogueira et al. (2017)
United Arab Emirates	3–6 or NA	2.2–6.9	84.5–91.1 or NA	26.4–29.9 or NA	NA	NA	5–28 or NA	NA	NA	NA	Anemia, septic meningitis (death), splenic sequestration crisis, repeated infections, brain infarction	el-Kalla and Mathews (1997)
Turkey	11	NA	NA	NA	NA	NA	NA	NA	NA	NA	Anemia, hepatosplenomegaly, mild jaundice, moderate vasoocclusive crises	Jiskoot et al. (2004)
India	0.3–60	2.3–14.1 or NA	76.1–111.6 or NA	24.1–36.8 or NA	0–59.3 or NA	0–3.8 or NA	1.1–29.9 or NA	9.8–45.5 or NA	7–52.4 or NA	2.8 or NA	Pale, upper respiratory tract infection, anemia with sickle cell, cerebral ischaemic infarcts, sudden onset painless diminution of vision, fever, hepatosplenomegaly and globular abdomen, joint problems, painful crises, multiple foci of osteonecrosis, jaundice, acute chest syndrome, gall stones, avascular necrosis of bilateral femoral head, vaso-occlusive crisis, moderate pain in both extremities, mild abdominal pain, loss of appetite, painful vaso-occlusive crisis, puffiness of face	Singh et al. (2023), Shanthala Devi et al. (2016), Rahimah et al. (2014), Afzal and Umair (2016), Gangwe et al. (2023), Shah et al. (2022), Oberoi et al. (2014), Archana et al. (2022), Patel et al. (2014), Italia et al. (2014), Sahiba et al. (2012)
Greece	2	8.5	84.3	27.9	NA	NA	NA	NA	NA	NA	Transient migrating arthriti, upper respiratory tract infection, jaundice, mild anemia, pallor	Athanasiou-Metaxa et al. (2002)
Iraq	15^a^	6.3–9.2	94.8–99.6	32–35.1	NA	4.1 or NA	14.3–18	42.3 or NA	35.1 or NA	NA	Fatigue, bone pain, anemia, painful crises, splenic sequestration crisis, repeated chest infections, hepatosplenomegaly	el-Kalla and Mathews (1997), Lund et al. (2015)
Kuwait	8–24	NA	NA	NA	NA	NA	22–26.5	44.5–48.5	24.7–30.7	NA	Pallor, acute splenic sequestration, vaso-occlusive crisis, acute osteomyelitis, acute chest syndrome, upper quadrant pain, avascular bone necrosis	Adekile et al. (2010)
African-American	NA	8.2	73	NA	0	0	0	30	53	17^b^	Abdominal pain	Husan et al. (2022)
Pakistan	6–10^a^	9.3–10.6	82.9–91.2	27.5–30.7	NA	NA	7–32	NA	NA	NA	Asymptomatic	el-Kalla and Mathews (1997)
Mexican	4–14 or NA	6.4–8.5	85.3–96	28.1–30.5 or NA	NA	1.75–2.99	2.47–4.92	NA	NA	NA	Splenomegaly, hepatomegaly	Perea et al. (1999)
Hb D-Punjab + β-thalassemia, heter												
India	0.66–56	2.5–13.8 or NA	48.1–78.3 or NA	14.2–23.8 or NA	0–49.6 or NA	2.3–6.3	0.9–15 or NA	75–88.2	NA	13.3^c^ or NA	Progressive generalised weakness, easy fatigability, pale, anemia, icteric with splenomegaly, reduced activity levels, awareness of mass in abdomen, headache, abdomen distention, recurrent upper respiratory tract infection with fever, severe pneumonia, asymptomatic or NA	Shanthala Devi et al. (2016), Singh et al. (2023), Shekhda et al. (2017), Pandey et al. (2012), Adekile et al. (1996), Waye et al. (2024), Ballas et al. (1977), Sharma et al. (2020), Kumaresan et al. (2011), Kaur et al. (2018)
Afghan	6	11.5	51.4	16.5	3.50	4.70	<0.5	91.8	NA	NA	Mild anemia with erythrocytosis	Huits et al. (2022)
English	36	12.7	66	21.1	0	7.2	1.4	91	NA	NA	Suspected glandular fever, and the symptoms subsided in due course	Worthington and Lehmann (1985)
kuwait	4.5–39	11.3–14	55.7–64.5	17.3–19.8	0	5.3–5.6	2.9–3.5	90.9–91.8	NA	NA	Weight and height were on the 50th percentile on the growth chart	Adekile et al. (1996)
Mexican	75	9	57.4	17.8	NA	NA	1.9	NA	NA	NA	Asymptomatic	Perea et al. (1999)
Thailand	7–18	11–14.8	52.5–67.1	18.1–22.1	5.1–9.8	3.2–5.3	4.9 or NA	80.7–86.3	NA	NA	Asymptomatic or NA	Panyasai et al. (2017), Fucharoen et al. (2002)
United Arab Emirates	1^a^-40 or NA	11–13.4	59.3–62.9	18.9–20.1	0–1.26	1.19–4.7	0.03–14 or NA	80–96	NA	NA	Asymptomatic or NA	el-Kalla and Mathews (1997), Belhoul et al. (2013)
Pakistani	12–50^a^	9.8–12.6	58.6–60.6	18.6–18.7	0	4.9–5.4	0.6–1	94	NA	NA	Asymptomatic	el-Kalla and Mathews (1997)
Iran	3–40^a^ or NA	10.7–14.8	54.5–68.9	17.7–23.1 or NA	0–30 or NA	4–6.8	0–18.1	59–94.5	NA	16.8–21.4 or NA	Asymptomatic or NA	el-Kalla and Mathews (1997), Rahimi et al. (2006), Taghavi Basmanj et al. (2011)
Brazil	1	9.2	61	19	33.8	4.4	4.3	57.5	NA	NA	Mild anemia	Zago and Costa (1988)
Greece	32	13.9	67.3	21.7	17.9	2.3	0.8	79	NA	NA	Asymptomatic	Theodoridou et al. (2009)

^a^ Combined with α-thalassemia.

^b^ Combined with Hb Korle thus had an Hb Korle-Bu peak.

^c^ Combined with Hb Q-India thus had an Hb Q-India peak. Hb X, uncharacterized hemoglobin peak.

## Discussion

In this study, the SMRT sequencing was employed to conduct a population-based genetic screening for hemoglobinopathies among large-scale individuals all over Guangxi, China. The hemoglobin variants, Hb O-Arab and Hb D-Punjab at the same genetic locus, were identified in two unrelated families, with an extremely low frequency among the screened population. Furthermore, we predicted the impact of these two rare variants on the structure of hemoglobin, and conducted a literature review to analyze their origins and genotype-phenotype correlations.

Hb O-Arab was first described in 1960 (Ramot et al., 1960), and was then discovered to cause the substitution of glutamic acid by lysine at residue 122 (Baglioni and Lehmann, 1962). Worldwide, Hb O-Arab was most common in the Pomak village in Greece with an allele frequency of 4.4% (Kuchenbaecker et al., 2022). Its frequency increased due to high genetic drift within the Pomak population, leading to its dispersion throughout the Mediterranean basin and the Middle East, with minor variations in its haplotypic pattern (Papadopoul et al., 2005). To date, Hb O-Arab had been reported in Israeli Arabs (Rachmilewitz et al., 1985), Tunisia (Kalai et al., 2024; Moumni et al., 2011; Nagel et al., 1999), Morocco (Morlé et al., 1984), and Bulgaria (Kantchev et al., 1975). However, to the best of our knowledge, this was the first reported family with the Hb O-Arab variant in the Chinese population, among whom it was expected to have an extremely low occurrence. It was believed that the Hb O-Arab variant originated from the Ottoman Empire, as the high-incidence regions for Hb O-Arab were consistent with the areas colonized by the Ottoman Empire (Elbashir and Elsayed Yousif, 2023). Few clues could be found to support the origin of the variant in this Chinese family. Despite this, the identification of the variant expanded the mutational spectrum of the *HBB* gene in the Chinese population. It should be declared that this variant had not been included in the routine screening panels in most clinical settings in China, and therefore the true frequency of the variant in the population may have been higher than anticipated. Additionally, heterozygous carriers of the Hb O-Arab variant were clinically asymptomatic and could have been easily neglected in routine screening, resulting in an unusually low detection rate.

By reviewing the reported cases in the literature, we found highly variable phenotypes among individuals carrying the Hb O-Arab variant. The characteristics of Hb O-Arab homozygotes varied from asymptomatic with only mild anemia to mild symptoms, including jaundice, splenomegaly, lassitude, anorexia, and epigastric pain (Efremov et al., 1977; Dror, 2013). The phenotypes of compound heterozygotes for Hb O-Arab and Hb S were similar to those of homozygotes for Hb S disease, presenting with hemolytic anemia, jaundice, and sickle cell disease characteristics (Rachmilewitz et al., 1985). The compound heterozygotes for Hb O-Arab and the β-thalassemia variant typically manifested mild to moderate anemia, and the hematological characteristics and clinical manifestations of individuals with Hb O-Arab combined with β^0^-thalassemia individuals were considerably more severe than those of individuals with Hb O-Arab combined with β^+^-thalassemia individuals (Moumni et al., 2011; Morlé et al., 1984; Kantchev et al., 1975; Rachmilewitz et al., 1985; Kalai et al., 2024), indicating that β-thalassemia variants were the main contributors to the phenotypic variability in these individuals. A previous study reported a four-month-old infant with compound heterozygous Hb O-Arab and β^0^-thalassemia variant (*HBB*: c.92 + 1G>A), presenting severe manifestations, including neonatal hemolytic anemia and an enlarged spleen (Kalai et al., 2024). However, the proband in Family 1 that we reported did not present with microcytic hypochromic anemia combined with abnormal hemoglobin content, increased bilirubin, icteric sclera, and skin discoloration until the age of 18, suggesting variable severity and onset age of the manifestations in these cases.

Hb D-Punjab, also known as Hb D-Los Angeles, was one of the most common hemoglobin variants worldwide, following Hb S and Hb C. It was most prevalent in India, and also had been found in other countries, including Italy, Spain, Thailand, and so on (Torres et al., 2015). In China, Hb D-Punjab was most common in Xinjiang province, accounting for 55.6% of total abnormal hemoglobin variants (Li et al., 1986). The Hb D-Punjab variant had been reported in both heterozygous and homozygous states as well as in combination with other abnormal hemoglobins such as thalassemia or Hb S. Hb D-Punjab heterozygotes and homozygotes, the rarest form of inheritance, presented no clinical or hematological alterations, but occasionally manifested mild to moderate hemolytic anemia. Usually, the interaction between Hb D-Punjab and β-thalassemia led to mild microcytic and hypochromic anemia, but did not present relevant clinical or hematological changes. However, the co-inheritance of Hb S and D-Punjab resulted in moderate to severe clinical manifestations similar to those of homozygous Hb S. In this study, we observed a heterozygous Hb D-Punjab in the proband from Family 2, who was expected to be asymptomatic. However, the genetic counseling and carrier screening for his partner in the future are still necessary due to the increased risk of having offspring with compound heterozygosity for Hb S and Hb D-Punjab.

The selection of strategies for diagnosing hemoglobinopathies is largely determined by the variant spectrum and prevalence of the variants in the local population. In China, the conventional methods of genetic testing for thalassemia, such as Gap-PCR and PCR-RDB (Polymerase Chain Reaction-Reverse Dot Blot), typically detect only the 24 hotspot variants commonly found in the Chinese population, which cover approximately 95%–98% of α- and β-thalassemia carriers (Li and Ye, 2024). However, various rare and complex variants are booming with global migration. Population-specific assays can not fully satisfy the needs of hemoglobinopathies controlling and prevention, thus optimizing diagnostic strategies and improving testing rates are essential. Compared with conventional methods, the SMRT approach provides the ability to uncover new variations and complex structural rearrangements such as triplications of the α-globin genes which worsen β-thalassemia phenotypes with high efficiency benefiting from its free of PCR amplification during sequencing and use of ultra-long reads (Xu et al., 2024). Furthermore, it can distinguish whether the identified variants are in *cis* or *trans* configuration without pedigree analysis (Xu et al., 2020). Recently, the SMRT sequencing has emerged as a reliable method for preconception screening and prenatal testing of hemoglobinopathies (Huang et al., 2024; Liang et al., 2023). Integrating this approach into screening programs for newborns in high-incidence areas who are at high risk of hemoglobinopathies could enhance early diagnosis, enable personalized treatment, support informed decision-making, and ultimately improve public health outcomes for these conditions.

## Conclusion

Two rare hemoglobin variants, Hb O-Arab and Hb D-Punjab, were identified in the population-based genetic screening throughout Guangxi, China, using the SMRT sequencing. The first report of Hb O-Arab enriches the spectrum of hemoglobin variants in the Chinese population. Analyzing the frequency, origin, and genotype-phenotype correlation of these variants could pave the way for clinical management and genetic counseling for hemoglobinopathies, including thalassemia. Due to the limited number of subjects enrolled and the complex genotype-phenotype correlation involved, further research based on a larger volume of participants in specific populations is still required to summarize the key hematological and clinical features of these rare variants. This study also verified the SMRT sequencing-based assay as a valuable and comprehensive method for the detection of rare hemoglobin variants.

## Data Availability

The original contributions presented in the study are included in the article/supplementary material, further inquiries can be directed to the corresponding authors.

## References

[B1] Adekile A. D. Kazanetz E. G. Leonova J. Y. Marouf R. Khmis A. Huisman T. H. (1996). Co-inheritance of Hb D-Punjab (codon 121; GAA--CAA) and beta (0) -thalassemia (IVS-II-1; G--A). J. Pediatr. Hematology/Oncology 18 (2), 151–153. 10.1097/00043426-199605000-00010 8846127

[B2] Adekile A. Mullah-Ali A. Akar N. A. (2010). Does elevated hemoglobin F modulate the phenotype in Hb SD-Los Angeles? Acta Haematol. 123 (3), 135–139. 10.1159/000276998 20110664

[B3] Afzal H. Umair S. F. (2016). Haemoglobin sickle D disease: a presentation with ischaemic stroke. JPMA J. Pak. Med. Assoc. 66 (3), 348–350. 26968293

[B4] Ali W. Jain M. Agarwal S. Kumar A. (2020). A case of hemoglobin sickle-D Punjab. Indian J. Hematol. Blood Transfus. 36 (1), 205–207. 10.1007/s12288-019-01179-6 32158109 PMC7042435

[B5] Archana R. Vidya C. Sumithra N. Jyothi M. Sanil R. (2022). Arab-Indian -530 ß-distal promoter haplotype and sickle/Hb D heterozygosis in Badagas of Nilgiris: is it suggestive of Harappan origin? J. Genet. 101, 17. 10.1007/s12041-021-01348-5 35221311

[B6] Athanasiou-Metaxa M. Economou M. Tsatra I. Pratsidou P. Tsantali C. (2002). Co-inheritance of hemoglobin D-Punjab and hemoglobin S: case report. J. Pediatr. Hematology/Oncology 24 (5), 421. 10.1097/00043426-200206000-00022 12142798

[B7] Baglioni C. Lehmann H. (1962). Chemical heterogeneity of haemoglobin O. Nature 196, 229–232. 10.1038/196229a0 13968953

[B8] Ballas S. K. Atwater J. Norris D. G. (1977). The interaction of betaO-thalassemia with hemoglobin D Punjab: a study of globin chain synthesis in an Indian family. Int. J. Hemoglobin Res. 1 (7), 697–701. 10.3109/03630267708999176 914644

[B9] Belhoul K. Bakir M. L. Abdulrahman M. J. H. (2013). Misdiagnosis of Hb D-Punjab/β-Thalassemia is a potential pitfall in hemoglobinopathy screening programs: a case report. Hemoglobin 37, 119–123. 10.3109/03630269.2013.769174 23425159

[B10] Biswas T. K. Pillai A. (2019). Hemoglobin-D Punjab—rare hemolytic anemia in the elderly: a case report. MGM J. Med. Sci. 6 (3), 148–151. 10.4103/mgmj.mgmj_12_20

[B11] Desai D. V. Dhanani H. Shah M. A. Dayal N. Kapoor A. Yeluri S. (2003). Homozygous hemoglobin D disease: a case report. Internet J. Pathology 3. 10.5580/1a8b

[B12] Dror S. (2013). Clinical and hematological features of homozygous hemoglobin O-Arab [beta 121 Glu → Lys]. Pediatr. Blood Cancer 60 (3), 506–507. 10.1002/pbc.24414 23192960

[B13] Efremov G. D. Sadikario A. Stojancov A. Dojcinov D. Huisman T. H. (1977). Homozygous hemoglobin O Arab in a gypsy family in Yugoslavia. Hemoglobin 1 (4), 389–394. 10.3109/03630267708996897 893136

[B14] el-Kalla S. Mathews A. R. (1997). Hb D-Punjab in the United Arab Emirates. Hemoglobin 21 (4), 369–375. 10.3109/03630269709000669 9255615

[B15] Elbashir I. Elsayed Yousif T. Y. (2023). Molecular detection of hemoglobin O-Arab in the Sudanese population. Int. J. General Med. 16, 3323–3330. 10.2147/IJGM.S421140 PMC10406112 37554704

[B16] Fucharoen S. Changtrakun Y. Surapot S. Fucharoen G. Sanchaisuriya K. (2002). Molecular characterization of Hb D-Punjab [beta121(GH4)Glu--Gln] in Thailand. Hemoglobin 26 (3), 261–269. 10.1081/hem-120015030 12403491

[B17] Gangwe A. B. Singh A. Parchand S. M. Agrawal D. Ekumankama C. B. Azad R. (2023). Asymmetric sickle cell retinopathy in a patient with sickle cell hemoglobin D disease: a case report. Indian J. Ophthalmol. 3 (3), 760–761. 10.4103/ijo.ijo_981_23

[B18] Giardine B. M. Joly P. Pissard S. Wajcman H. Chui D. H. K. Hardison R. C. (2021). Clinically relevant updates of the HbVar database of human hemoglobin variants and thalassemia mutations. Nucleic Acids Res. 49 (D1), D1192–D1196. 10.1093/nar/gkaa959 33125055 PMC7778921

[B19] Harteveld C. L. Achour A. Arkesteijn S. J. G. Ter Huurne J. Verschuren M. Bhagwandien-Bisoen S. (2022). The hemoglobinopathies, molecular disease mechanisms and diagnostics. Int. J. Lab. Hematol. 44 (Suppl. 1), 28–36. 10.1111/ijlh.13885 36074711 PMC9542123

[B20] Heard S. E. Westwood N. B. Pearson T. C. Stephens A. D. (1991). Homozygous haemoglobin O-Arab in pregnancy. Clin. Laboratory Haematol. 13 (3), 319–320. 10.1111/j.1365-2257.1991.tb00289.x 1794236

[B21] Huang R. Liu Y. Xu J. Lin D. Mao A. Yang L. (2024). Back-to-Back comparison of third-generation sequencing and next-generation sequencing in carrier screening of thalassemia. Archives Pathology Laboratory Med. 148 (7), 797–804. 10.5858/arpa.2022-0168-OA 36630651

[B22] Huits R. Feyens A. M. Lonneville N. Peyrassol X. Adam A.-S. Gulbis B. (2022). Diagnosis and clinical relevance of co-inheritance of haemoglobin D-Punjab/β+-thalassemia traits in an immigrant Afghan family. J. Clin. Pathol. 75, 861–864. 10.1136/jclinpath-2021-208009 35039448 PMC9685697

[B23] Husan A. Amarasinghe S. N. Fontenot A. Khan M. W. (2022). Rare combinational hemoglobinopathies. Cureus 14 (12), e32327. 10.7759/cureus.32327 36628031 PMC9825142

[B24] Italia K. Upadhye D. Dabke P. Kangane H. Colaco S. Sawant P. (2014). Clinical and hematological presentation among Indian patients with common hemoglobin variants. Clin. Chim. Acta 431, 46–51. 10.1016/j.cca.2014.01.028 24508621

[B25] Jiskoot P. M. Halsey C. Rivers R. Bain B. J. Wilkins B. S. (2004). Unusual splenic sinusoidal iron overload in sickle cell/haemoglobin D-Punjab disease. J. Clin. Pathology 57 (5), 539–540. 10.1136/jcp.2002.004481 PMC1770290 15113864

[B26] Kalai M. Moumni I. Ouragini H. Chaouechi D. Boudriga I. Menif S. (2024). Coinheritance of HbO Arab/β0-thalassemia with severe manifestation in newborn. Am. J. Perinatology 41 (5), 594–597. 10.1055/s-0042-1743185 35189650

[B27] Kantchev K. Tcholakov B. Casey R. Lehmann H. El Hazmi M. J. H. (1975). Twelve families with Hb O Arab in the Burgas district of Bulgaria. Observations on sixteen examples of Hb O Arab-beta (0) thalassaemia. Humangenetik 26, 93–97. 10.1007/BF00278434 1112610

[B28] Kaur M. Bodal V. K. Sibia R. P. S. Gupta D. Murarka S. (2018). Education m. A rare presentation of haemoglobin D-thalassemia. J. Res. Med. Edu 18 (1). 10.56412/GMCP.2018.1.1.13

[B29] Kohne E. (2011). Hemoglobinopathies: clinical manifestations, diagnosis, and treatment. Dtsch. Arzteblatt Int. 108 (31-32), 532–540. 10.3238/arztebl.2011.0532 PMC3163784 21886666

[B30] Kountouris P. Lederer C. W. Fanis P. Feleki X. Old J. Kleanthous M. (2014). IthaGenes: an interactive database for haemoglobin variations and epidemiology. PLoS One 9 (7), e103020. 10.1371/journal.pone.0103020 25058394 PMC4109966

[B31] Kuchenbaecker K. Gilly A. Suveges D. Southam L. Giannakopoulou O. Kilian B. (2022). Insights into the genetic architecture of haematological traits from deep phenotyping and whole-genome sequencing for two Mediterranean isolated populations. Sci. Rep. 12 (1), 1131. 10.1038/s41598-021-04436-9 35064169 PMC8782863

[B32] Kumaresan K. Gupta K. Kalra N. Das R. (2011). A rare association of giant adrenal myelolipoma in a young female double heterozygous for HbD Punjab and β-thalassemia trait. Indian J. Pathol. Microbiol. 54 (3), 635–637. 10.4103/0377-4929.85126 21934247

[B33] Lacerra G. Fioretti G. Hani A. Duka D. De Angioletti M. Pagano L. (1993). Hb O-Arab [beta 121(GH4)Glu--Lys]: association with DNA polymorphisms of African ancestry in two Mediterranean families. Hemoglobin 17 (6), 523–535. 10.3109/03630269309043492 7908281

[B34] Lai K. Huang G. Su L. He Y. (2017). The prevalence of thalassemia in mainland China: evidence from epidemiological surveys. Sci. Rep. 7 (1), 920. 10.1038/s41598-017-00967-2 28424478 PMC5430438

[B35] Li W. Ye Y. (2024). Application of third-generation sequencing technology in the genetic testing of thalassemia. Mol. Cytogenet. 17 (1), 32. 10.1186/s13039-024-00701-4 39696632 PMC11657128

[B36] Li H. J. Liu D. X. Li L. Liu Z. G. Lo S. L. Zhao J. (1986). A note about the incidence and origin of Hb D-Punjab in Xinjiang, People's Republic of China. Hemoglobin 10 (6), 667–671. 10.3109/03630268609036571 3557998

[B37] Liang Q. Gu W. Chen P. Li Y. Liu Y. Tian M. (2021). A more universal approach to comprehensive analysis of thalassemia alleles (CATSA). J. Mol. Diagnostics JMD. 23 (9), 1195–1204. 10.1016/j.jmoldx.2021.06.008 34293487

[B38] Liang Q. He J. Li Q. Zhou Y. Liu Y. Li Y. (2023). Evaluating the clinical utility of a long-read sequencing-based approach in prenatal diagnosis of thalassemia. Clin. Chem. 69 (3), 239–250. 10.1093/clinchem/hvac200 36683393

[B39] Lund K. Chakravorty S. Toma S. Bain B. J. (2015). Compound heterozygosity for hemoglobins S and D. Am. J. Hematol. 90 (9), 842. 10.1002/ajh.24095 26103542

[B40] Modell B. Darlison M. (2008). Global epidemiology of haemoglobin disorders and derived service indicators. Bull. World Health Organ. 86 (6), 480–487. 10.2471/blt.06.036673 18568278 PMC2647473

[B41] Morlé F. Morlé L. Baklouti F. Dorléac E. Baudonnet C. Delaunay J. (1984). The Hb F composition in a Moroccan family with β°‐thalassaemia and Hb O‐Arab. Scand. J. Haematol. 33 (3), 281–287. 10.1111/j.1600-0609.1984.tb02229.x 6209785

[B42] Moumni I. Yalaoui S. Ghrairi N. Hamzaoui A. Zoraï A. Abbes S. (2011). Molecular characterization of a discrete hemoglobinopathy upon investigation for a lung hydatic cyst in an old Tunisian patient. Ann. Biol. Clin. Paris. 69 (3), 353–356. 10.1684/abc.2011.0582 21659055

[B43] Nagel R. L. Krishnamoorthy R. Fattoum S. Elion J. Genard N. Romero J. (1999). The erythrocyte effects of haemoglobin O(ARAB). Br. J. Haematol. 107 (3), 516–521. 10.1046/j.1365-2141.1999.01755.x 10583251

[B44] Nikolov N. Andreeva M. Janković L. Efremov G. D. (1989). Hemoglobin O Arab in interaction with beta 0-thalassemia. Lijec. Vjesn. 111 (1-2), 27–30. 2739498

[B45] Nogueira R. N. Leite CMBT Souza L. X. Barbosa A. A. L. (2017). Clinical and laboratory repercussions in patient with hemoglobin SD-Punjab disease: a case report. J. Bras. Patol. Med. Lab. 53 (5). 10.5935/1676-2444.20170049

[B46] Oberoi S. Das R. Trehan A. Ahluwalia J. Bansal D. Malhotra P. (2014). HbSD-Punjab: clinical and hematological profile of a rare hemoglobinopathy. J. Pediatr. Hematology/Oncology 36 (3), e140–e144. 10.1097/MPH.0000000000000049 24276032

[B47] Pandey S. Ranjan R. Mishra R. M. Pandey S. Saxena R. (2012). Interaction of - α 3.7, ß thalassemia mutation IVS 1-5 and HbD Punjab in a family: a case report. Indian J. Clin. Biochem. IJCB 27 (3), 314–317. 10.1007/s12291-012-0189-8 26405395 PMC4577505

[B48] Panyasai S. Rahad S. Pornprasert S. (2017). Coinheritance of hemoglobin D-Punjab and β(0)-thalassemia 3.4 kb deletion in a Thai girl. Asian J. Transfus. Sci. 11 (2), 199–202. 10.4103/ajts.AJTS_117_16 28970692 PMC5613431

[B49] Papadopoulos V. Dermitzakis E. Konstantinidou D. Petridis D. Xanthopoulidis G. Loukopoulos D. (2005). HbO-Arab mutation originated in the Pomak population of Greek Thrace. Haematologica 90 (2), 255–257. 15710581

[B50] Patel S. Purohit P. Mashon R. S. Dehury S. Meher S. Sahoo S. (2014). The effect of hydroxyurea on compound heterozygotes for sickle cell-hemoglobin D-Punjab--a single centre experience in eastern India. Pediatr. Blood Cancer 61 (8), 1341–1346. 10.1002/pbc.25004 24616059

[B51] Perea F. J. Casas-Castañeda M. Villalobos-Arámbula A. R. Barajas H. Alvarez F. Camacho A. (1999). Hb D-Los Angeles associated with Hb S or beta-thalassemia in four Mexican Mestizo families. Hemoglobin 23 (3), 231–237. 10.3109/03630269909005703 10490135

[B52] Politis-Tsegos C. Kynoch P. Lang A. Lehmann H. Lorkin P. A. Stathopoulou R. (1975). Homozygous haemoglobin D Punjab. J. Med. Genet. 12 (3), 269–274. 10.1136/jmg.12.3.269 1177278 PMC1013288

[B53] Rachmilewitz E. A. Tamari H. Liff F. Ueda Y. Nagel R (1985). The interaction of hemoglobin O Arab with Hb S and beta+ thalassemia among Israeli Arabs. Hum. Genet. 70, 119–125. 10.1007/BF00273069 3859465

[B54] Rahimah A. Syahira Lazira O. Siti Hida H. M. Faidatul Syazlin A. H. Nur Aisyah A. Nik Hafidzah N. M. (2014). Haemoglobin sickle d Punjab: - a case report. Med. J. Malays. 69 (1), 42–43. 24814631

[B55] Rahimi Z. Akramipour R. Korani S. Nagel R. L. (2006). Hb D-Punjab [beta 121 (GH4) Glu--Gln]/beta0-thalassemia [IVSII.1(G--A)] in two cases from an Iranian family: first report. Am. J. Hematol. 81 (4), 302–303. 10.1002/ajh.20537 16550524

[B56] Ramot B. Fisher S. Remez D. Schneerson R. Kahane D. Ager J. A. (1960). Haemoglobin O in an Arab family. Br. Med. J. 2 (5208), 1262–1264. 10.1136/bmj.2.5208.1262 20788973 PMC2097055

[B57] Rezende Pdo V. Costa K. S. Domingues J. C. Silveira P. B. Belisário A. R. Silva C. M. (2016). Clinical, hematological and genetic data of a cohort of children with hemoglobin SD. Rev. Bras. Hematol. Hemoter. 38 (3), 240–246. 10.1016/j.bjhh.2016.05.002 27521862 PMC4997897

[B58] Richards S. Aziz N. Bale S. Bick D. Das S. Gastier-Foster J. (2015). Standards and guidelines for the interpretation of sequence variants: a joint consensus recommendation of the American college of medical genetics and genomics and the association for molecular pathology. Genet. Med. 17 (5), 405–424. 10.1038/gim.2015.30 25741868 PMC4544753

[B59] Sahiba K. Amandeep K. Namrata C. (2012). Hemoglobin SD disease - a case report. Biochem. 6 (7), 223–225.

[B60] Shah V. Shah M. Jhaveri P. Shah F. (2022). Double heterozygosity of Hbs and Hbd Punjab in two siblings with Covid-19 infection: a case report. Indian J. Appl. Basic Med. Sci. 24, 18–23. 10.48165/ijabms.2022.243803

[B61] Shanthala Devi A. M. Rameshkumar K. Sitalakshmi S. (2016). Hb D: a not so rare hemoglobinopathy. Indian J. Hematol. Blood Transfus. 32 (Suppl. 1), 294–298. 10.1007/s12288-013-0319-3 27408416 PMC4925467

[B62] Sharma P. Jandial A. Rajasekaran S. Das R. Chhabra S. Hira J. K. (2020). Missing Hb Q-India peak in a triple-heterozygous patient with Hb D-Punjab/Hb Q-India/β-Thalassemia trait. Hemoglobin 44 (3), 211–213. 10.1080/03630269.2020.1767128 32448026

[B63] Shekhda K. M. Leuva A. C. Mannari J. G. Ponda A. V. Amin A. (2017). Co-inheritance of haemoglobin D-Punjab and beta thalassemia - a rare variant. J. Clin. Diagn. Res. 11 (6), OD21–OD22. 10.7860/JCDR/2017/27816.10114 28764232 PMC5535424

[B64] Silva-Pinto A. C. Silva T. J. Moretto E. L. Ottoboni M. Rodrigues E. S. Covas D. T. (2014). Blood donor homozygous for Hb D Los Angeles. Transfus. Apher. Sci. 51 (2), 219–220. 10.1016/j.transci.2014.08.021 25217459

[B65] Singh N. Seth T. Tyagi S. (2023). Review of clinical and hematological profile of hemoglobin D cases in a single centre. J. Mar. Med. Soc. 25, S74–S79. 10.4103/jmms.jmms_165_22

[B66] Spandana R. Panneerselvam K. Mani S. Krishnamoorthy N. (2022). An interesting and rare case of hemoglobin D-Punjab variant in Tamil Nadu. Cureus 14 (2), e22668. 10.7759/cureus.22668 35371765 PMC8965195

[B67] Taghavi Basmanj M. Karimipoor M. Amirian A. Jafarinejad M. Katouzian L. Valaei A. (2011). Co-inheritance of hemoglobin D and β-thalassemia traits in three Iranian families: clinical relevance. Arch. Iran. Med. 14 (1), 61–63. 21194265

[B68] Theodoridou S. Alemayechou M. Perperidou P. Sinopoulou C. Karafoulidou T. Kiriakopoulou G. (2009). Compound heterozygosity for Hb D-Punjab/β-thalassemia and blood donation: case report. Turk J. Hematol. 26 (2), 100–101. 27265282

[B69] Torres L. S. Okumura J. V. Silva D. G. Bonini-Domingos C. R. (2015). Hemoglobin D-Punjab: origin, distribution and laboratory diagnosis. Rev. Bras. Hematol. Hemoter. 37 (2), 120–126. 10.1016/j.bjhh.2015.02.007 25818823 PMC4382585

[B70] Torres L. S. Okumura J. V. Belini-Júnior É. Oliveira R. G. Nascimento P. P. Silva D. G. (2016). Phenotypic diversity of sickle cell disease in patients with a double heterozygosity for Hb S and Hb D-Punjab. Hemoglobin 40 (5), 356–358. 10.1080/03630269.2016.1222295 27535451

[B71] van Gammeren A. J. Pelkmans L. Endschot C. Roelofsen-de Beer R. Harteveld C. L. (2020). An unusual compound heterozygosity for Hb O-Arab (HBB: c.364G>A) and Hb D-Los Angeles (HBB: c.364G>C). Hemoglobin 44 (1), 61–63. 10.1080/03630269.2019.1710530 31973650

[B72] Waye J. S. Hanna M. Hohenadel B. A. Nakamura L. Walker L. Eng B. (2024). β(0)-Thalassemia caused by a novel nonsense mutation [HBB: c.199A > T]. Hemoglobin 48 (1), 69–70. 10.1080/03630269.2024.2322518 38425097

[B73] Weatherall D. J. (2010). The inherited diseases of hemoglobin are an emerging global health burden. Blood 115 (22), 4331–4336. 10.1182/blood-2010-01-251348 20233970 PMC2881491

[B74] Worthington S. Lehmann H. (1985). The first observation of Hb D Punjab beta zero thalassaemia in an English family with 22 cases of unsuspected beta zero thalassaemia minor among its members. J. Med. Genet. 22 (5), 377–381. 10.1136/jmg.22.5.377 4078867 PMC1049482

[B75] Xu L. Mao A. Liu H. Gui B. Choy K. W. Huang H. (2020). Long-molecule sequencing: a new approach for identification of clinically significant DNA variants in α-thalassemia and β-thalassemia carriers. J. Mol. Diagnostics JMD. 22 (8), 1087–1095. 10.1016/j.jmoldx.2020.05.004 32473995

[B76] Xu A. Ye Y. Huang Y. Huang Y. Guo H. Ji L. (2024). Identification of Hb Lepore, Hb anti-Lepore, and α-globin gene triplications by long-read single-molecule real-time sequencing. Am. J. Clin. Pathology 161 (4), 411–417. 10.1093/ajcp/aqad155 38037185

[B77] Zago M. A. Costa F. F. (1988). Hb D-Los Angeles in Brazil: simple heterozygotes and associations with beta-thalassemia and with Hb S. Hemoglobin 12 (4), 399–403. 10.3109/03630268808998040 3170242

[B78] Zimmerman S. A. O'Branski E. E. Rosse W. F. Ware R. E. (1999). Hemoglobin S/O(Arab): thirteen new cases and review of the literature. Am. J. Hematol. 60 (4), 279–284. 10.1002/(sici)1096-8652(199904)60:4<279::aid-ajh5>3.0.co;2-2 10203101

